# Reactive oxygen species and redox compartmentalization

**DOI:** 10.3389/fphys.2014.00285

**Published:** 2014-08-12

**Authors:** Nina Kaludercic, Soni Deshwal, Fabio Di Lisa

**Affiliations:** ^1^Neuroscience Institute, National Research Council of Italy (CNR)Padova, Italy; ^2^Department of Biomedical Sciences, University of PadovaPadova, Italy

**Keywords:** reactive oxygen species, compartmentalization, mitochondria, oxidative stress, redox signaling

## Abstract

Reactive oxygen species (ROS) formation and signaling are of major importance and regulate a number of processes in physiological conditions. A disruption in redox status regulation, however, has been associated with numerous pathological conditions. In recent years it has become increasingly clear that oxidative and reductive modifications are confined in a spatio-temporal manner. This makes ROS signaling similar to that of Ca^2+^ or other second messengers. Some subcellular compartments are more oxidizing (such as lysosomes or peroxisomes) whereas others are more reducing (mitochondria, nuclei). Moreover, although more reducing, mitochondria are especially susceptible to oxidation, most likely due to the high number of exposed thiols present in that compartment. Recent advances in the development of redox probes allow specific measurement of defined ROS in different cellular compartments in intact living cells or organisms. The availability of these tools now allows simultaneous spatio-temporal measurements and correlation between ROS generation and organelle and/or cellular function. The study of ROS compartmentalization and microdomains will help elucidate their role in physiology and disease. Here we will examine redox probes currently available and how ROS generation may vary between subcellular compartments. Furthermore, we will discuss ROS compartmentalization in physiological and pathological conditions focusing our attention on mitochondria, since their vulnerability to oxidative stress is likely at the basis of several diseases.

## Introduction

Reactive oxygen species (ROS) formation and redox signaling are well known to play a major role in physiology as well as in a variety of pathologies. For instance, in the heart, cardiomyocyte differentiation, and excitation-contraction coupling are under tight redox control (Burgoyne et al., 2012; Steinberg, 2013). On the other hand, cardiac pathologies, such as ischemia/reperfusion injury, heart failure, and arrhythmias can be prevented or blocked by inhibiting specific processes that result in ROS generation in several experimental models (Takimoto and Kass, 2007; Youn et al., 2013; Anderson et al., 2014; Kaludercic et al., 2014b). Thus, it appears that pro-oxidant generation and antioxidant defense need to be tightly regulated (Chance et al., 1979). Indeed, disruption of redox signaling and control, and imbalance in favor of pro-oxidant species is defined oxidative stress, term first coined in 1985 (Sies, 1985; Sies and Cadenas, 1985; Jones, 2006). Conversely from pathological modifications (Chance et al., 1979; Powers and Jackson, 2008), it appears that physiological redox signaling is characterized by reversible oxido-reductive modifications, confined both spatially and temporally in subcellular compartments and microdomains.

To exert their effects, ROS have to induce a reversible change that results in the modification of protein activity. The first step is the single-electron oxidation of a thiol to a thiyl radical, which can then react to form disulphide bonds with glutathione (GSH) or with another protein thiol (Wardman and Von Sonntag, 1995; Collins et al., 2012). Nevertheless, thiols can be further oxidized by ROS and result in higher oxidation states of sulfur (Steinberg, 2013). Such changes have limited or no reversibility under biological conditions (Jones and Go, 2010; Steinberg, 2013).

Quantification of thiol oxidation on cellular and subcellular levels has shown that thiol/disulphide couples such as GSH and thioredoxin (Trx) are maintained at stable values and are not in equilibrium relative to each other in different organelles (Go and Jones, 2008; Jones and Go, 2010). This suggests that redox status as a consequence of ROS production is not necessarily a global imbalance of oxidative and reductive processes, but rather that thiol oxidation in different cellular compartments serves as means for cell signaling, protein trafficking and regulation of enzyme, receptor, transporter and transcription factor activity (Balaban et al., 2005; D'Autreaux and Toledano, 2007). This consideration, termed the “redox hypothesis,” postulates that oxidizable thiols are control elements organized in redox circuits that are physically and kinetically separated so that they are highly responsive and can function independently to regulate different biological processes (Jones and Go, 2010). However, upon a certain threshold in ROS formation, these circuits can be disrupted. Indeed, the occurrence of oxidative stress overwhelms the cellular antioxidant defense and results in lack of control over redox signaling mechanisms. These concepts can now be validated employing new redox sensors that allow dynamic and compartmentalized ROS measurements and their correlation with organelle/cell function and viability.

Thus, ROS generation within specific subcellular compartments and their redox status appear to be of major importance for understanding cell pathophysiology. Recently, new methods for the study of redox compartmentalization have become available. This is a rapidly growing field that led to the development of, and was then contributed by, probes that now permit observation of rapid redox changes in real time and with single organelle resolution not only in live cells, but also in living animals (Woolley et al., 2013; Ezerina et al., 2014; Lukyanov and Belousov, 2014). Here we will review the tools currently available for the measurement of ROS and redox potential within single organelles and discuss the data available so far on ROS compartmentalization in physiological and pathological conditions focusing our attention on mitochondria as the major source and target of ROS.

## Tools to study compartment redox status and ROS formation

In order to study the relationship between ROS formation and cell (dys)function, it is necessary to define which species are produced, in what amount and to characterize them in a spatio-temporal manner. Redox potential of a specific compartment or cell can be studied using a variety of techniques to identify and quantify major redox couples or redox sensitive proteins within organelles. For instance, high-performance liquid chromatography (HPLC) is used for the quantification of GSH/GSSG and NAD(P)H/NAD(P)^+^ redox potentials (Jones, 2002; Takimoto et al., 2005), whereas mass spectrometry and redox Western blotting, in association with labeling of free thiols, are frequently used to determine the redox state of several proteins such as Trx, Trx reductase, and others (Halvey et al., 2005; Chen et al., 2006; Go et al., 2009; Go and Jones, 2013). Although these methods present high specificity for the redox couple examined and both the oxidized and reduced form can be quantified, they often require tissue/cell fractionation, during which redistribution and artifactual oxidation/reduction can occur. To overcome these problems molecular biology techniques using epitope-tagged versions of nuclear localization sequence (NLS)-Trx-1 and nuclear export sequence (NES)-Trx1 (specifically localized in nuclei or cytoplasm) have been developed that allow measurements without fractionation (Go et al., 2010). More recent approaches involve fluorescent imaging techniques of ROS and major redox couples within organelles in intact cells or organisms *in vivo*. Currently available fluorescent sensors for compartmentalized ROS detection can be divided into small molecule probes and genetically encoded fluorescent proteins. The overview of the methods presented here is by no means exhaustive and for in depth coverage the reader is referred to the following excellent reviews (Meyer and Dick, 2010; Go and Jones, 2013; Lukyanov and Belousov, 2014; Winterbourn, 2014).

### Small molecule redox probes

Although several small molecule fluorescent probes are available, only a few of them can be targeted to specific subcellular compartments (Table 1). It should be also mentioned that, to some extent, all these probes present limitations in terms of selectivity and sensitivity.

**Table 1:** **Small molecule fluorescent redox sensitive probes and their characteristics**.

**Redox sensor**	**Compartment**	**Excitation wavelength, nm**	**Emission wavelength, nm**	**Detected ROS**	**System used**	**References**
MitoSOX Red	Mitochondria	(396)510	580	O^•−^_2_	Intact cells	Robinson et al., 2006
MitoTracker Red CM-H_2_XRos	Mitochondria	579	599	Not specific	Intact cells	Poot et al., 1996
MitoTracker Orange CM-H_2_TMRos	Mitochondria	554	576	Not specific	Intact cells	Kweon et al., 2001
Peroxy Lucifer 1 (PL1)	Cytosol	410	475/540	H_2_O_2_	Intact cells	Srikun et al., 2008
Nuclear Peroxy Emerald 1 (NucPE1)	Nuclei	468/490	530	H_2_O_2_	Intact cells, *in vivo*	Dickinson et al., 2011
Mitochondrial Peroxy Yellow 1 (MitoPY1)	Mitochondria	510	528	H_2_O_2_	Intact cells	Dickinson et al., 2013
SHP-Mito	Mitochondria	342/383	470/545	H_2_O_2_	Intact cells	Masanta et al., 2012
MitoBoronic acid (MitoB)	Mitochondria	Mass spectroscopy		H_2_O_2_	*In vivo*	Cocheme et al., 2011; Logan et al., 2014

MitoSOX Red is widely used for measurement of superoxide formation in the mitochondria of live cells (Robinson et al., 2006; Zhou et al., 2011a). MitoSOX Red indicator is a derivative of hydroethidine (HE) and contains the cationic triphenylphosphonium substituent that is responsible for the electrophoretically driven uptake of the probe in actively respiring mitochondria. The reaction between superoxide and HE generates a highly specific red fluorescent product, 2-hydroxyethidium. Nevertheless, another red fluorescent product, ethidium, can be formed from other oxidants in biological systems (Zhao et al., 2005). Thus, a simple fluorescence assay cannot distinguish between superoxide and other oxidants. The superoxide-specific product can be detected by HPLC or mass spectrometry and only then it provides a reliable method for superoxide production (Zhao et al., 2005; Zielonka et al., 2009).

Reduced MitoTracker dyes, MitoTracker Orange CM-H_2_TMRos, and MitoTracker Red CM-H_2_XRos, are derivatives of dihydrotetramethyl rosamine and dihydro-X-rosamine, respectively. These reduced probes become fluorescent and positively charged upon their oxidation in live cells, and thus accumulate in mitochondria according to the Nernst equation (Poot et al., 1996; Kweon et al., 2001; Kaludercic et al., 2014a). As with MitoSOX Red, the quick and easy loading into the cells makes these probes very convenient. However, reduced MitoTracker dyes are not specific for single oxidant species and the fact that their accumulation is dependent on the mitochondrial membrane potential may lead to artifactual measurements.

In order to overcome problems associated with oxidant sensitive dyes, new generation of fluorescent probes has been developed. These are often referred to as “non-redox” probes as they contain a masked fluorophore that is released by the attack of the oxidant on the blocking group, without changing the oxidation state of the fluorophore (Winterbourn, 2014). The boronate derivatives, i.e., sensors that have boronate as blocking group, have been synthesized for the detection of hydrogen peroxide (H_2_O_2_) (Miller et al., 2005). Nevertheless, it was shown that some boronate probes also respond to peroxynitrite and hypochlorous acid, thus raising some concerns regarding their specificity (Sikora et al., 2009). Peroxy Green1 (PG1) and Peroxy Crimson1 (PC1) are second-generation probes that are sensitive enough to report H_2_O_2_ production at physiological signaling levels while maintaining H_2_O_2_ specificity and are activated by a single boronate deprotection (Miller et al., 2007). Because of their enhanced turn-on responses to H_2_O_2_, these new chemical tools are capable of detecting endogenous bursts of H_2_O_2_ produced by growth factor signaling in living cells (Miller et al., 2007; Lin et al., 2013). Nevertheless, these probes were not targeted to a specific compartment. There is a wide range of compounds with different fluorophores (Dickinson et al., 2010) and adapted structures to enable targeting to mitochondria (Dickinson et al., 2013) and other compartments, such as nuclei and endoplasmic reticulum (ER) (Srikun et al., 2008; Dickinson et al., 2011; Woolley et al., 2012). In particular, combining boronate-phenol chemistry with mitochondria-targeting functional group, such as positively charged phosphonium moiety, led to generation of Mitochondrial Peroxy Yellow (MitoPY1), SHP-Mito, and MitoBoronic acid (MitoB) (Cocheme et al., 2012; Masanta et al., 2012; Dickinson et al., 2013). SHP-Mito is also a ratiometric probe and allows for increased penetration depth and prolonged imaging time using two-photon microscopy (Masanta et al., 2012). MitoB instead is a ratiometric mass spectrometry probe that is rapidly converted to phenol product MitoP upon H_2_O_2_ oxidation (Cocheme et al., 2011). Measurement of MitoB/MitoP ratio has been successfully used *in vivo* and allows accurate measurements of H_2_O_2_ in the nanomolar range (Cocheme et al., 2011; Logan et al., 2014).

### Fluorescent protein based redox sensors

Due to the need to overcome problems and limitations of conventional redox measurements related to specificity, reversibility, quantitation, and subcellular targeting, genetically encoded redox sensitive probes based on fluorescent proteins were developed (Table 2). This represents a major breakthrough, since these probes present high redox species specificity, their oxidation is reversible thus allowing dynamic real-time measurements and, importantly, can be targeted to specific subcellular compartments.

**Table 2:** **Genetically encoded fluorescent protein based redox sensors and their characteristics**.

**Redox sensor**	**Compartment**	**Excitation wavelength, nm**	**Emission wavelength, nm**	**Detected ROS**	**System used**	**References**
**FLUORESCENT PROTEIN BASED REDOX SENSORS**						
rxYFP	Nuclei, cytosol, mitochondrial matrix, IMS	512	527	GSH/GSSG	Intact cells	Ostergaard et al., 2004; Hu et al., 2008; Banach-Latapy et al., 2013
roGFP1	Mitochondria, IMS, cytosol, nuclei, ER	400/480	510	GSH/GSSG	Intact cells	Dooley et al., 2004; Hanson et al., 2004; Van Lith et al., 2011; Birk et al., 2013; Rodriguez-Rocha et al., 2013
roGFP2	Cytosol, mitochondria, ER, nuclei	400/495	515	GSH/GSSG	Intact cells, *in vivo*	Dooley et al., 2004; Guzman et al., 2010; Birk et al., 2013
cpYFP	Mitochondria	405/488	515	O^•−^_2_	Intact cells, *in vivo*	Wang et al., 2008; Fang et al., 2011; Shen et al., 2014
**REDOX SENSITIVE FLUORESCENT PROTEINS COUPLED TO REDOX ACTIVE ENZYMES**						
Grx1-roGFP2	Cytosol, mitochondria, IMS, ER, nuclei	405/488	515	GSH/GSSG	Intact cells, *in vivo*	Gutscher et al., 2008; Albrecht et al., 2011; Kojer et al., 2012; Birk et al., 2013; Breckwoldt et al., 2014
Orp1-roGFP2	Mitochondria, cytosol	405/488	515	H_2_O_2_	Intact cells, *in vivo*	Albrecht et al., 2011
HyPer	Cytosol, mitochondria, IMS, ER, peroxisomes, nuclei	420/488	515	H_2_O_2_	Intact cells, *in vivo*	Malinouski et al., 2011; Mishina et al., 2011; Lukyanov and Belousov, 2014
rxYFP-Grx1		512	523	GSH/GSSG	*In vitro*	Bjornberg et al., 2006
**REDOX SENSITIVE FLUORESCENT PROTEINS FUSED TO MEMBRANE PROTEINS**						
EGFR-HyPer	Plasma membrane, endosomes	420/488	515	H_2_O_2_	Intact cells	Mishina et al., 2011
PDGFR-HyPer	Plasma membrane, endosomes	420/488	515	H_2_O_2_	Intact cells	Mishina et al., 2011
HyPer-TA	Cytoplasmic side of ER membrane	420/488	515	H_2_O_2_	Intact cells	Mishina et al., 2011
p47phox-roGFP2	Plasma membrane	400/495	515	H_2_O_2_	Intact cells, *in vivo*	Pal et al., 2013
DuoxA1-OxyFRET	Plasma membrane	435	535/480	H_2_O_2_	Intact cells	Enyedi et al., 2013
DuoxA1-PerFRET	Plasma membrane	435	535/480	H_2_O_2_	Intact cells	Enyedi et al., 2013
roGFP1-Lamp2a, roGFP1-Cnx, roGFP1-CD63, TrfR-roGFP1	Lysosomes, ER, endosomes	400/480	510	GSH/GSSG	Intact cells	Austin et al., 2005
**FRET BASED REDOX SENSORS**						
OxyFRET	Mitochondria, cytosol, plasma membrane	435	535/480	H_2_O_2_	Intact cells	Enyedi et al., 2013
PerFRET	Mitochondria, cytosol, plasma membrane	435	535/480	H_2_O_2_	Intact cells	Enyedi et al., 2013
HSP-FRET	Cytosol	430	470/535	Not specific	Intact cells	Waypa et al., 2006; Robin et al., 2007
Organic Hydroperoxide Sensor (OHSer)	Cytosol, nuclei	519	526	H_2_O_2_	Intact cells	Zhao et al., 2010, 2013
**NADPH SENSORS**						
Frex	Mitochondria, cytosol, nuclei	420/500	535	NADH	Intact cells	Zhao et al., 2011
Peredox	Cytosol, mitochondria	400	510	NADH	Intact cells	Hung et al., 2011

Initially, green fluorescent protein (GFP)-based redox sensitive proteins were developed introducing cysteine residues onto fluorescent protein scaffolds (Ostergaard et al., 2001). The redox state of these cysteines equilibrates with the GSH/GSSG ratio in a process catalyzed by the thiol-disulfide exchanging enzyme glutaredoxin (Grx), and leads to changes in chromophore spectra upon oxidation or reduction. Indeed, the redox sensitive yellow fluorescent protein (rxYFP) allows for non-invasive quantitative imaging of the dithiol-disulfide equilibrium (Ostergaard et al., 2001, 2004; Hu et al., 2008; Banach-Latapy et al., 2013). However, rxYFP is an intensiometric rather than a ratiometric probe. Moreover, its equilibration in different redox states depends on Grx availability and is very slow, thus representing a rate-limiting factor. This limitation was overcome fusing rxYFP to Grx1 (rxYFP-Grx1), rendering it independent of host organism Grx availability (Bjornberg et al., 2006).

Introduction of cysteines into fluorescent proteins led to the generation of redox sensitive GFP (roGFP). Initially, roGFP1 was developed that provided ratiometric fluorescence readout and increased sensitivity compared to rxYFP (Dooley et al., 2004; Hanson et al., 2004). Among the 6 roGFP variants available at present, roGFP2 has the highest dynamic range and has been best characterized (Meyer and Dick, 2010). Moreover, imaging of roGFP2 is easier, since the anionic form of the chromophore (presenting stronger fluorescence) decreases and the protonated form (initially showing lower fluorescence) increases upon oxidation, whereas the opposite is true for roGFP1 (Lukyanov and Belousov, 2014).

Generation of a redox relay between the redox sensing domain and redox sensitive fluorescent protein provided both specificity and efficiency to the redox sensing process. This idea was further used to improve roGFPs available. roGFP2 was fused to Grx1 (Grx1-roGFP2) and responds to either GSH or GSSG in a time scale of minutes and senses redox potential changes between −240 and −320 mV (Gutscher et al., 2008). When incubated with H_2_O_2_, Grx1-roGFP2 was insensitive to oxidation and only addition of GSH to the mixture led to the oxidation of the sensor, indicating its high specificity for the GSH redox status. It is important to note that, although roGFPs contain cysteine residues close to the chromophore, these cysteines do not present high redox reactivity, influencing the oxidation state of the probe only upon enzymatic oxidation. roGFPs can be targeted to several compartments of the cell, such as cytosol, mitochondria, intermembrane space (IMS), ER, nucleus, lysosomes, and endosomes (Dooley et al., 2004; Waypa et al., 2010; Albrecht et al., 2011; Van Lith et al., 2011; Birk et al., 2013), or fused to specific proteins to immobilize it in the cell (Pal et al., 2013). In terms of applicability, conventional redox sensing fluorescent proteins such as rxYFP and roGFPs are more suitable for the measurement of steady state redox conditions, since the kinetics of complete intracellular equilibration of the probe with the GSH system is slow and can take tens of minutes (Meyer and Dick, 2010). On the other hand, chimeric fusion proteins of redox active enzymes and redox sensitive fluorescent proteins facilitate rapid and complete equilibration with a defined cellular redox couple. Indeed, Grx1-roGFP2 is currently the probe of choice for dynamic measurements of GSH/GSSG ratio in subcellular compartments (mitochondria, IMS, cytosol) in a variety of experimental settings, from intact cells to animals *in vivo* (Gutscher et al., 2008; Albrecht et al., 2011; Kojer et al., 2012; Breckwoldt et al., 2014).

The same group also developed Orp1-roGFP2 by fusing roGFP2 with yeast peroxidase Orp1 (Gutscher et al., 2009). This resulted in an H_2_O_2_-sensitive probe that is pH insensitive, ratiometric, and reports submicromolar concentrations of H_2_O_2_. This sensor has been targeted to the cytosol or to the mitochondria and allows dynamic H_2_O_2_ measurements both *in vitro* and *in vivo* (Albrecht et al., 2011).

Another H_2_O_2_ sensitive sensor with similar characteristics is HyPer, developed inserting the circularly permuted YFP (cpYFP) into the regulatory domain of *E. coli* H_2_O_2_ sensing protein OxyR (Choi et al., 2001). HyPer demonstrates specificity and submicromolar affinity to H_2_O_2_, and can be targeted to the cytosol, mitochondria, IMS, ER, peroxisomes, or nuclei (Malinouski et al., 2011). As with roGFPs, the advantages of HyPer are reversibility (it can be reduced by cellular thiol-reducing systems), and the possibility to perform ratiometric measurements thus preventing imaging artifacts caused by object movement or differences in expression levels between cells or compartments (Lukyanov and Belousov, 2014). However, one concern that deserves attention is the influence of pH on HyPer fluorescence. The excitation fluorescence ratio is significantly shifted by pH (even by a small shift of 0.2 pH units) and could lead to artifactual results (Meyer and Dick, 2010). Thus, when using HyPer it is necessary to monitor pH changes in the same compartment [for example using pH-indicator SypHer, a mutated form of HyPer generated mutating one of the two H_2_O_2_-sensing cysteine residues of the OxyR domain (Poburko et al., 2011)]. Indeed, the same problem of pH sensitivity occurs with cpYFP, initially developed as a superoxide sensor targeted to the mitochondrial matrix (Wang et al., 2008), making it unclear whether the observed changes are due to superoxide or pH changes. Recently, two enhanced versions of HyPer have been developed, HyPer-2 and -3, with HyPer-3 showing an expanded dynamic range and higher brightness upon expression in cells while maintaining the oxidation and reduction rates fast (Markvicheva et al., 2011; Bilan et al., 2013). HyPer has also been targeted to the plasma membrane and specific loci by fusing it to the epidermal growth factor receptor (EGFR), platelet derived growth factor receptor (PDGFR) or to the tail anchor sequence of the protein tyrosine phosphatase 1B (PTP-1B) (Mishina et al., 2011). The same has been done also with the roGFP2 that has been fused to the NADPH oxidase (Nox) organizer protein p47phox (Pal et al., 2013) making it possible to readily detect H_2_O_2_ close to the source of its production.

Due to their recent development, limited information is available on fluorescence resonance energy transfer (FRET) based redox sensors. Initially, a linker containing two cysteine residues was placed between enhanced cyan and yellow fluorescent protein FRET pair, but showed a too small dynamic range of the probes to be used for imaging (Kolossov et al., 2008). Other novel FRET probes were recently developed (OxyFRET, PerFRET, HSP-FRET, rOHSer) (Waypa et al., 2006; Robin et al., 2007; Zhao et al., 2010, 2013; Enyedi et al., 2013), and targeted to mitochondria, cytoplasm, nuclei or plasma membrane or they were fused to the dual oxidase (Duox) activator DuoxA1 to achieve colocalization with Duox1 (Enyedi et al., 2013). Nevertheless, more studies are necessary to fully characterize these sensors. For instance, OxyFRET contains N- and C-terminal cysteine rich domains of Yap1 as H_2_O_2_ sensitive regions, which relies on the peroxidase function of Orp1. Since Orp1 is not present in mammalian cells, it remains to be elucidated which cellular redox couples or enzymes are actually responsible for the oxidation of the probe (Enyedi et al., 2013).

Finally, two genetically encoded sensors for NADH (Frex, Peredox) were developed placing circularly permuted fluorescent proteins (cpFPs) into a tandem dimer of bacterial Rex protein, capable of binding NADH (Hung et al., 2011; Zhao et al., 2011). Incorporation of cpFP into a linker between two Rex subunits results in Rex dimerization upon NADH binding and change in fluorescence. These sensors demonstrate highly specific affinity for NADH and do not respond to NADH analogs, including NADPH. While the Frex sensor is based on cpYFP and thus requires pH control (Zhao et al., 2011), Peredox is pH-stable, but intensiometric (Hung et al., 2011). Moreover, the extremely high sensitivity of Peredox to NADH precludes its use in the mitochondrial matrix where the NADH/NAD^+^ ratio is high. Nevertheless, mitochondrial redox status can also be monitored measuring NADH/flavin ratio through their autofluorescence, an approach based on the pioneering work by Britton Chance in the 50's (Chance and Baltscheffsky, 1958; Chance and Jobsis, 1959). The great advantage of this approach is the rapid response to stimuli and its minimal invasiveness, which permit mitochondrial redox status monitoring not only in intact cells, but also in different organs *in vivo* (Chance et al., 1962; Mayevsky and Chance, 1973, 1982). Indeed, technological advances in this field led to the development of devices for the measurement of NADH/flavin ratio and tissue vitality also in patients (Mayevsky et al., 1996; Mayevsky and Rogatsky, 2007).

### Limitations

One of the major issues in live cell imaging is phototoxicity, which occurs upon repeated exposure of fluorescently labeled cells to illumination. In their excited state, fluorescent molecules tend to react with molecular oxygen to produce free radicals that can damage subcellular components and compromise the entire cell. Phototoxicity depends on several variables: (1) photochemical properties of the fluorescent protein, (2) its concentration and subcellular localization, (3) the excitation intensity. Importantly, the total excitation light dose should be kept to minimum and it is preferable to use probes with longer wavelength excitation light, since excitation at a shorter wavelength is more damaging to cells because of increased efficiency of ROS production (Dailey et al., 2006). Thus, the imaging process requires optimization in order to find the right balance between image quality and light induced damage that may alter cell physiology (Dixit and Cyr, 2003).

Fluorescent proteins are generally not phototoxic to cells, due to the fact that their fluorophores are buried deep within a polypeptide envelope. Nevertheless, it is not possible to exclude that alterations in the cell physiology occur due to introduction of a ~30 kDa protein as a fluorescent sensor or that the presence of a redox sensitive probe may interfere with the physiological redox signaling. In addition, tissue and cellular oxygen distribution may also play a role in ROS detection, since it is not homogeneous (Williams et al., 2012; Lloyd et al., 2014). These concerns need to be taken into account, and, when possible, appropriate controls should be performed.

## Compartmentalization of ROS formation and redox signals in physiology and pathology

In recent years it has become increasingly clear that oxidative and reductive modifications are confined in a spatio-temporal manner. This makes ROS signaling similar to that of Ca^2+^ (Rizzuto and Pozzan, 2006; Brasen et al., 2010; Petersen, 2014) or other second messengers, such as cyclic adenosine monophosphate (cAMP) (Stangherlin and Zaccolo, 2012). Some subcellular compartments are more oxidizing (such as ER, lysosomes, or peroxisomes) whereas others are more reducing (mitochondria, nuclei). Although more reducing, mitochondria are especially susceptible to oxidation, most likely due to the high number of exposed thiols present in that compartment (Jones and Go, 2010). Here we will examine how ROS generation may vary between subcellular compartments and determine beneficial effects or lead to pathology.

### Mitochondria

Mitochondria are considered as the most redox-active compartment in the cell accounting for more than 90% of oxygen utilization. Although the vast majority of oxygen undergoes complete reduction to water at the level of cytochrome oxidase, partial reduction accompanied by ROS generation can occur as well (Boveris and Chance, 1973). Several other mitochondrial proteins, such p66^Shc^ and monoamine oxidases (MAOs) among others, are prominent sites for ROS generation (Kaludercic et al., 2014b). Nevertheless, mitochondria are apparently very well equipped with antioxidant defense systems and are capable of maintaining a high degree of GSH reduction under normal conditions (Hanson et al., 2004; Jones and Go, 2010). This tight control of the mitochondrial redox status might be interpreted as a mechanism of protection, since a low rate of ROS generation is a normal process in mitochondria whereas excess can lead to cell death. Indeed, mitochondria are very susceptible to oxidation and considered to be both the source and target of ROS. Moreover, an initial ROS burst from the mitochondria may trigger a process termed as “ROS-induced ROS release” from neighboring mitochondria, amplifying oxidative stress, and leading to cell death (Zorov et al., 2000).

Mitochondria play a key role in energy metabolism and thus, depending on the cell and tissue type, may act as nutrient and oxygen sensors. Ca^2+^ uptake by mitochondria tightly regulates cellular metabolism by stimulating the activity of several key dehydrogenases (Denton, 2009). The finely tuned interplay between mitochondrial ROS, Ca^2+^, and glucose appears to be the regulatory mechanism for insulin release from pancreatic β-cells (Leloup et al., 2011; Maechler, 2013). Indeed, insulin release is stimulated by mitochondrial ROS in response to glucose and requires extracellular Ca^2+^ for this mobilization (Leloup et al., 2009). Moreover, ROS can promote insulin sensitivity through phosphatase and tensin homolog (PTEN) inactivation and phosphoinositide 3-kinase (PI3K)/Akt signaling (Loh et al., 2009). On the other hand, mitochondrial dysfunction, and enhanced ROS generation are associated with insulin resistance and thus, type 2 diabetes (Kim et al., 2008; Anderson et al., 2009; Szendroedi et al., 2012).

A recent study examining the fine line between mitochondrial redox signals in physiology and pathology was performed on mice expressing Grx1-roGFP2 sensor in neuronal mitochondria (Breckwoldt et al., 2014). A multiparametric *in vivo* imaging approach was used to assess mitochondrial function simultaneously measuring mitochondrial redox status, membrane potential, pH, and Ca^2+^ levels. Redox potential of axonal mitochondria was tightly regulated under physiological conditions, although individual mitochondria showed short-lived redox bursts followed by spontaneous and reversible changes in shape (contractions), rapid mitochondrial depolarization, and increase in pH. These changes were independent of mitochondrial Ca^2+^ levels, but highly dependent on mitochondrial ROS formation and were increased exposing axons to higher neuronal activity. This suggests that these reversible mitochondrial redox changes might serve as a signal to overcome acute challenges and protect from eventual damage. However, under pathological conditions, such as in a chronic amyotrophic lateral sclerosis model or axotomy, rapid mitochondrial Ca^2+^ increase led to long-lasting mitochondrial oxidation, irreversible changes in shape and opening of the permeability transition pore (PTP), suggesting that noxious levels of stress may induce more permanent, Ca^2+^-induced, mitochondrial derangements.

Another study employing cpYFP expressed in cardiomyocyte mitochondria similarly showed that individual mitochondria undergo spontaneous bursts of superoxide production (termed superoxide flashes), triggered by transient openings of the mitochondrial PTP (Wang et al., 2008; Fang et al., 2011). These flashes increased in frequency after stress, such as anoxia/reoxygenation, and were generated by the respiratory chain. Moreover, flash frequency in early adulthood was recently shown to negatively correlate with lifespan in *C. elegans* (Shen et al., 2014). Of note, none of these studies performed an adequate pH control and thus remain controversial, since, as discussed above, cpYFP is very sensitive to pH. Therefore, changes in fluorescence could have been caused by transient alkalinization of the mitochondrial matrix (Schwarzlander et al., 2011, 2012). Indeed, pH sensor SypHer (representing an adequate pH control for cpYFP) was able to detect similar mitochondrial flashes (Quatresous et al., 2012; Santo-Domingo et al., 2013) along with increase in MitoSOX fluorescence, which is stable at physiological pH. Therefore, more studies are needed to elucidate this controversy, but at the moment it cannot be excluded that actually both processes (superoxide and pH bursts) may take place.

Similar bursts in mitochondrial ROS formation were correlated with oscillations in organelle membrane potential and cardiomyocyte Ca^2+^ spark frequency under both physiological and pathological conditions (Aon et al., 2003, 2007, 2008; Zhou et al., 2011a) and were observed also in yeast cells, driven by the ultradian clock (Lloyd et al., 2003). Indeed, it has been proposed that individual, weakly coupled oscillating mitochondria are present in physiological conditions when ROS levels are low, but when the cellular redox status is perturbed, the mitochondrial network throughout the cell locks to one main low-frequency, high-amplitude oscillatory mode (Aon et al., 2008; Kurz et al., 2010). Transient changes in mitochondrial redox status, membrane potential, and uncoupling were also observed during normal autonomous pacemaking in dopaminergic neurons in the substantia nigra pars compacta (Guzman et al., 2010). Nevertheless, disruption of these processes compromised Ca^2+^-induced uncoupling and increased oxidation of matrix proteins, providing an explanation for their loss in Parkinson's disease. These studies suggest that transient changes in mitochondrial ROS, membrane potential, and shape might be part of a protective mechanism by which a signal is produced within a single organelle to isolate it from the rest of the mitochondrial network prompting its selective removal through mitophagy.

Mitochondrial ROS formation may also act as a signal to regulate organelle homeostasis. Hypoxia-inducible factor 1α (HIF1α), activated by hypoxia, is suggested to respond to and modulate mitochondrial oxidant production, through transcriptional regulation of several microRNAs that control the expression of components of the electron transport chain, lactate dehydrogenase A and PDK1 (pyruvate dehydrogenase kinase 1) (Guzy et al., 2005, 2008; Mansfield et al., 2005). Moreover, the increase of mitochondrial ROS needs to be conspicuous and diffuse to the cytosol in order to stabilize HIF1α suggesting that a certain threshold needs to be reached for this signaling to occur (Guzy et al., 2008). Increased mitochondrial ROS formation and HIF1α activation have also been proposed as the main mechanism involved in lifespan extension in *C. elegans* (Schulz et al., 2007; Lee et al., 2010b; Yang and Hekimi, 2010). Moreover, peroxisome proliferator-activated receptor gamma coactivator 1α (PGC1α), a transcription factor activated by mitochondrial ROS, HIF1α stabilization (O'Hagan et al., 2009), or through AMP-activated protein kinase (AMPK) stimulation (Canto et al., 2009), is required for the induction of many ROS-detoxifying enzymes, including GPx1 and superoxide dismutase 2 (SOD2), thus protecting the cell from oxidant induced death (St-Pierre et al., 2006).

A number of studies have evaluated ROS formation and redox status of the mitochondrial compartment in pathological conditions. Recent evidence points to mitochondrial oxidants as a signal for inflammasome activation (Bulua et al., 2011; Zhou et al., 2011b; Finkel, 2012). Moreover, oxidized mitochondrial redox state positively correlates with the metastatic potential and aggressiveness of breast cancer and melanoma (Li et al., 2009; Xu et al., 2010). Mitochondrial oxidative stress appears to precede ROS formation within other compartments and contributes to cell death during cardiomyocyte (Robin et al., 2007; Ranji et al., 2009; Loor et al., 2011), kidney (Hall et al., 2013) or liver ischemia (Haga et al., 2009) and vascular smooth muscle cells hypoxia (Desireddi et al., 2010; Waypa et al., 2010). Moreover, also other stimuli, such as parkinsonian toxins (Rodriguez-Rocha et al., 2013), metals (Hansen et al., 2006; Cheng et al., 2010) and nutrient deprivation (Go et al., 2007) can trigger mitochondrial oxidative stress. Tumor necrosis factor α (TNFα) also results in compartmentalized ROS formation in the mitochondria, Trx2 oxidation, downstream signaling to cytoplasm with nuclear factor kappa-light-chain-enhancer of activated B cells (NFκ B) activation and apoptosis (Hansen et al., 2004). In this study, cytosolic Trx1 was not oxidized, although more recent evidence employing mitochondria and cytosol targeted HyPer suggested that H_2_O_2_ increase was higher in the cytoplasm while mitochondrial matrix showed a lower response (Malinouski et al., 2011). Trx2 overexpression is protective against oxidant-induced apoptosis and several studies have shown that Trx2 is selectively susceptible to oxidative stress relative to cytoplasmic or nuclear Trx1 (Jones and Go, 2010), thus demonstrating the vulnerability of mitochondria to oxidative stress in a variety of cell types and pathologies (Kuroda et al., 2010; Stanley et al., 2011; Tocchetti et al., 2012).

**Figure 1:**
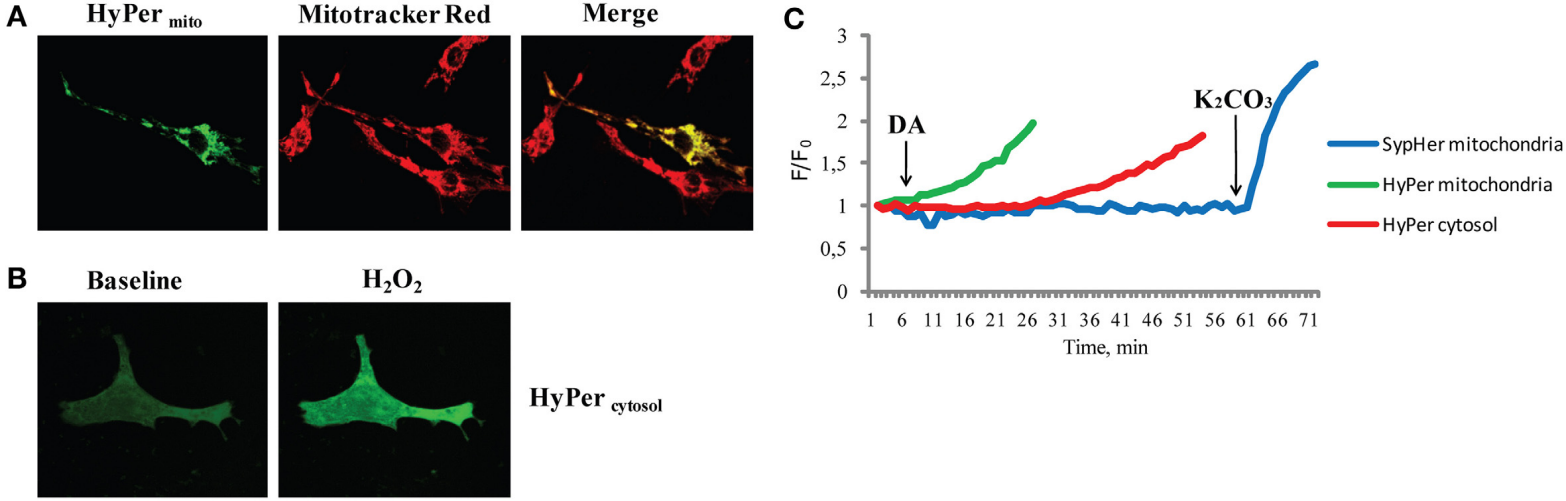
**H_2_O_2_ formation following MAO activation in different subcellular compartments. (A)** Neonatal rat ventricular cardiomyocytes transfected with genetically encoded mitochondria targeted H_2_O_2_ sensor HyPer (HyPer_*mito*_). Mitochondrial localization is confirmed by colocalization with the mitochondrial dye Mitotracker Red. **(B)** Neonatal cardiomyocytes expressing cytosol targeted HyPer (HyPer_cytosol_), at baseline and after the addition of H_2_O_2_ (100 μM). **(C)** The fluorescence ratio of mitochondria targeted HyPer (green line) increased shortly after the addition of MAO substrate dopamine (DA), wheras the cytosolic HyPer (red line) became oxidized only after prolonged incubation. In a separate experiment under the same conditions fluorescence of mitochondria targeted pH sensor SypHer (blue line) remained unchanged upon the addition of DA and increased only after matrix alkalinization with K_2_CO_3_. Reprinted by permission from Kaludercic et al. (2014a).

Mitochondrial ROS formation is of major importance also in cardiovascular diseases (Stowe and Camara, 2009; Camara et al., 2010; Kaludercic et al., 2011, 2014b). Several sites in mitochondria have been shown to generate ROS and thereby contribute to cardiac damage, but very few of them can be targeted pharmacologically and are therefore not suitable for therapy. Nevertheless, MAOs represent a promising therapeutic target. MAOs are flavoenzymes localized in the outer mitochondrial membrane responsible for neurotransmitter and biogenic amine catabolism. Recent work demonstrated that both MAO-A and -B activation results in mitochondrial ROS generation that promotes pathological hypertrophy and heart failure *in vivo*, cardiomyocyte death and ischemia/reperfusion injury (Bianchi et al., 2005; Pchejetski et al., 2007; Kaludercic et al., 2010, 2014a; Villeneuve et al., 2013). Inhibition of these enzymatic activities *in vivo* maintained cardiac function in pressure overloaded mice and prevented the transition to heart failure demonstrating once again the importance of mitochondrial ROS generation and its implication in pathology. Importantly, we demonstrated the existence of a direct link between MAO activation, mitochondrial ROS formation, and mitochondrial dysfunction (Kaludercic et al., 2014a). Using the H_2_O_2_ sensor HyPer targeted specifically to mitochondria or cytosol, ROS formation following MAO activation was analyzed in a spatio-temporal manner (Figure 1). We observed that H_2_O_2_ formation occurs much earlier at the mitochondrial level compared to the cytosol and was independent of pH, since SypHer fluorescence ratio remained unchanged under the same conditions. These redox changes were followed by the loss of mitochondrial membrane potential. This is an important finding, since it reiterates the issue that mitochondria are “early targets” of endogenously produced oxidative stress that leads to mitochondrial dysfunction. On the other hand, mitochondrial ROS can also trigger the activation of signaling cascades and transcription factors in other compartments, such as cytosol (as described above). In addition, mitochondrial ROS formation through MAOs was shown to lead to the oxidation of myofibrillar proteins in the failing heart, an event that negatively correlated with cardiac function (Canton et al., 2006, 2011).

### Cytoplasm

Besides being affected by mitochondrial ROS formation, physiological stimulation at the plasma membrane can cause the oxidation of specific redox couples in the cytosol without affecting other subcellular compartments. This is the case of EGFR activation that is accompanied by ROS formation, a critical component for proper signal transduction (Mishina et al., 2011). Indeed, EGFR activation led to the oxidation of cytoplasmic Trx1 redox potential by nearly 20 mV while nuclear and mitochondrial Trx2 and cellular GSH were not affected at all (Halvey et al., 2005).

Redox couples in the cytoplasm are not necessarily in equilibrium and the biggest advantage of this is the achievement of distinct signaling effects. Indeed, Trx1 and GSH/GSSG couples were found to vary independently during growth transitions, redox signaling, and metal induced toxicity (Go and Jones, 2008). Moreover, some studies reported that changes in GSH and H_2_O_2_ levels are highly restricted, not necessarily coupled and concurrent and H_2_O_2_ was found to positively correlate with life span in *D. melanogaster* (Albrecht et al., 2011). Others instead support the idea of pro-oxidative changes in association with aging and reduction in life span (Rebrin et al., 2004; Cocheme et al., 2011). These observations suggest that age dependent pro-oxidative changes are highly variable, restricted to particular tissues and underline the need to measure different oxidant species and redox couples separately and specifically in order to achieve complete biological information (Albrecht et al., 2011).

Oxidation occurring in the cytoplasmic compartment is frequently exploited as a signal that then triggers the translocation of proteins to other compartments to exert their function. One example of this mechanism is the redox dependent activation of transcription factors, such as NFκ B, activator protein 1 (AP-1) and NF-E2-related factor 2 (Nrf2) (Sen and Baltimore, 1986; Devary et al., 1991; Hansen et al., 2004). NFκ B activation occurs in the cytoplasm through Iκ B kinase mediated Iκ B phosphorylation, thus resulting in dissociation and release of NFκ B for its translocation into the nucleus (Sen and Baltimore, 1986). Nox1 overexpression led to an increased antioxidant response element (ARE) reporter gene expression mediated by H_2_O_2_-dependent c-Jun N terminal kinase (JNK) and extracellular signal-regulated kinases (ERK1/2) activation in the cytosol, but without affecting cytoplasmic GSH and Trx1 redox state (Go et al., 2004). This is likely due to the formation of localized increases in ROS formation following Nox activation that are not necessarily reflected globally in the whole cytoplasm (see below “ROS microdomains” section).

Thus, an increase in intracellular ROS as a result of exposure to a number of different stimuli can lead to oxidation of cysteine residues in cytoplasmic proteins, such as kinases and phosphatases, ultimately affecting signal transduction processes (Cumming et al., 2004). Indeed, one may envision cytoplasm as a “buffer zone” that allows for a low background of ROS used for sensitive and specific signaling (D'Autreaux and Toledano, 2007; Go and Jones, 2008). However, depending on the nature and duration of the insult, oxidation in the cytoplasmic compartment can also lead to cell death. For instance, Trx1 downregulation or oxidation results in AMPK oxidation and inactivation and apoptosis signal-regulating kinase 1 (ASK-1) activation, respectively, eventually leading to cell death (Liu et al., 2000; Shao et al., 2014).

### Nuclei

Nuclear redox couples (GSH, Trx1) are maintained at more reduced values than the ones in the cytoplasm, protecting the genome from ROS-induced damage. Indeed, many studies found oxidation in mitochondria and cytoplasm, but were unable to detect any changes in nuclei exposed to oxidants (Jones and Go, 2010). Nuclei contain GSH, which is critical for nuclear cysteine containing proteins. Trx1 translocates to the nucleus from cytoplasm in response to a variety of stimuli and its redox state then remains distinct from the one in the cytoplasm (Go and Jones, 2008). GSH is important for the regulation of nuclear matrix organization, maintenance of cysteine residues on zinc-finger DNA binding motifs in a reduced and functional state, chromosome consolidation, DNA synthesis, DNA protection from oxidative stress and protection of DNA-binding proteins (Go and Jones, 2008). Indeed, several transcription factors, including AP-1, NF-κ B, Nrf2, p53, and glucocorticoid receptor, contain a critical cysteine in the DNA binding region that is required for DNA binding (Go and Jones, 2008). These cysteine residues need to be in a reduced state in order to bind DNA and excessive ROS formation inhibits transcription factor—DNA binding (Abate et al., 1990; Toledano and Leonard, 1991; Hainaut and Milner, 1993; Bloom et al., 2002). Therefore, transcription factors can be activated in the cytoplasm through oxidation, but once in the nucleus their critical cysteine residues need to be reduced.

### Other compartments

Oxidizing environment in the ER lumen, required for introduction of structural disulfides during protein folding and secretion, is generated through continuous formation of GSSG in oxidative protein folding (Csala et al., 2006). The GSSG is kept within the ER compartment, since the ER membrane is impermeable to GSSG. Central redox proteins responsible for oxidative protein folding are Ero1p and protein disulfide isomerase (PDI). ER also contains a reductase system regulated by GSH and used to reduce incorrect protein disulfides (Chakravarthi et al., 2006). It is currently assumed that the redox state of the ER is optimally balanced for formation of disulfide bonds using GSH as the main redox buffer, and that unfolded protein response following induction of ER stress causes a reduction of this organelle whereby the release of misfolded protein is prevented (Enyedi et al., 2010; Delic et al., 2012; Birk et al., 2013). Indeed, this is the case with thapsigargin, although tunicamycin (another ER stress inducer) did not induce any reduction in ER of the HeLa cells (Birk et al., 2013). Therefore, the relationship between ER stress and its redox status warrants further investigation.

Peroxisomes are ubiquitous organelles involved in lipid metabolism and contain a number of enzymes that generate ROS and NO. Catalase is the major peroxisomal enzyme responsible for H_2_O_2_ metabolism and it appears that under physiological conditions H_2_O_2_ diffusion is prevented through its rapid conversion to O_2_. Interestingly, catalase expression is virtually absent in the peroxisomes of insulin-producing cells (Elsner et al., 2011). This lack of antioxidant defense impedes inactivation of peroxisome-generated H_2_O_2_ following fatty acid metabolism, thereby increasing the vulnerability of pancreatic β-cells to ROS-mediated lipotoxicity (Elsner et al., 2011). The importance of catalase in defense from oxidative damage is further supported by the beneficial effects afforded by targeting catalase expression in mitochondria or peroxisomes (Schriner et al., 2005; Dai et al., 2009, 2010; Lee et al., 2010a).

Both endosomes and lysosomes require reduction of disulfides for their function. Interestingly, a study measuring redox potentials of endocytic compartments by expressing roGFP fused to various endocytic proteins found that recycling endosomes, late endosomes, and lysosomes are oxidizing compartments, mimicking conditions in the ER (Austin et al., 2005). It could not be excluded that a minor subset of lysosomes could be reducing or that there could be subregions of reducing potential, implying that this topic deserves further investigation.

**Figure 2:**
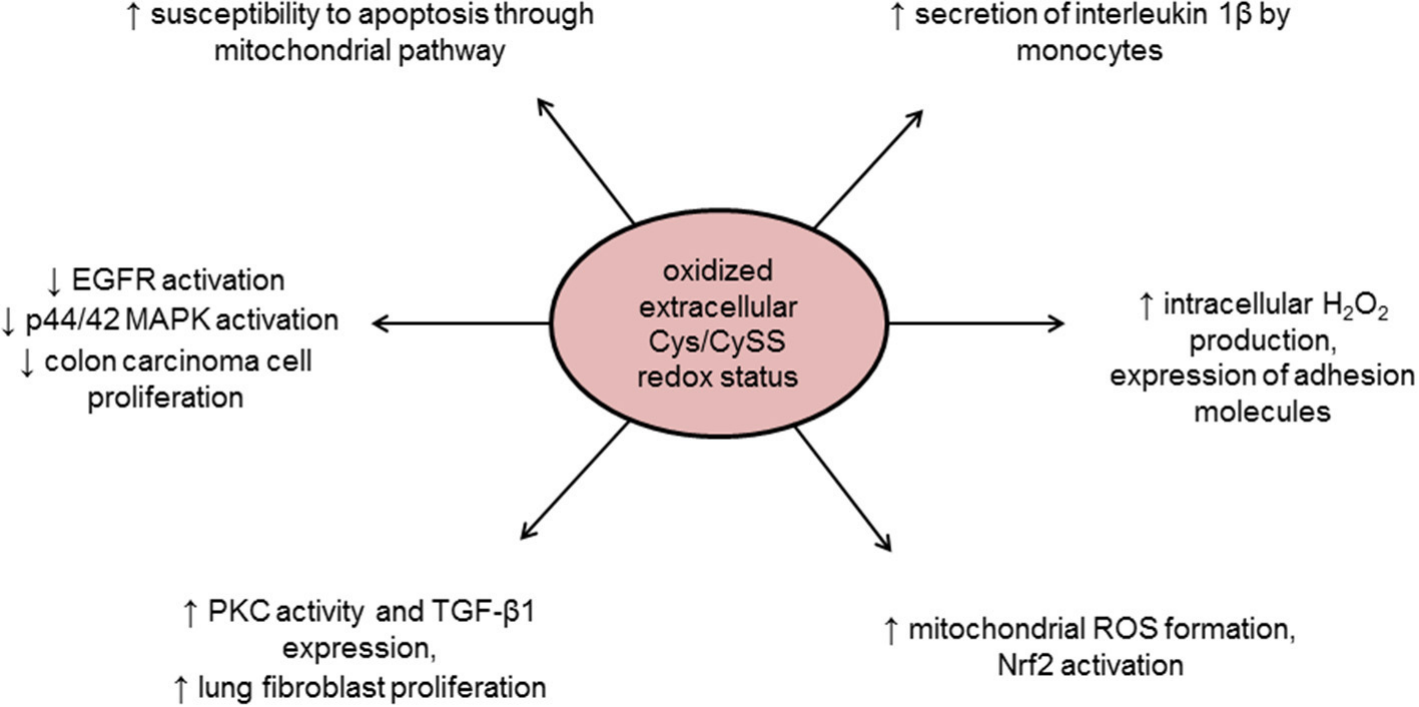
**Intracellular effects of oxidized extracellular redox conditions**. Cys/CySS, cysteine/cystine ratio; EGFR, epidermal growth factor receptor; MAPK, Mitogen-activated protein kinase; Nrf2, NF-E2-related factor 2; PKC, protein kinase C; ROS, reactive oxygen species; TGF-β1, transforming growth factor beta 1.

Finally, the extracellular redox state also plays an important role in the redox homeostasis. The GSH/GSSG, Trx1, and Cys/Cyss couples are relatively oxidized in extracellular space as compared to the cytoplasm, the latter being quantitatively the most significant redox couple in the extracellular space (Go and Jones, 2008; Banerjee, 2012). Indeed, extracellular Cys/CySS redox couple oxidation or reduction differently regulates cell growth, an effect that is cell type dependent and mediated by intracellular kinase activation (Nkabyo et al., 2005; Ramirez et al., 2007). Plasma redox potentials are oxidized in association with age, chemotherapy, diabetes, cardiovascular disease, and smoking (Go and Jones, 2008; Banerjee, 2012; Menazza et al., 2014). Moreover, extracellular redox status can also sensitize cells to oxidant-induced apoptosis through mitochondrial compartment (Jiang et al., 2005) and/or induce intracellular signal transduction (Figure 2). For instance, an increase in oxidized Cys/CySS redox status in the extracellular space can trigger mitochondrial ROS formation mediated by redox potential sensitive plasma membrane and cytoskeletal proteins involved in inflammation (Go et al., 2010). Oxidized cysteine redox potential has also been shown to increase the secretion of the pro-inflammatory interleukin 1β (Iyer et al., 2009), suggesting that pro-inflammatory effects of oxidized plasma redox couples might be due to a mitochondrial signaling pathway (Go et al., 2010). In addition, oxidizing extracellular environment may lead to mitochondrial ROS formation that in turn activates Nrf2 to up-regulate antioxidant and detoxification systems, although the exact mechanism linking oxidizing extracellular conditions and mitochondrial ROS formation was not described (Imhoff and Hansen, 2009).

### ROS microdomains

**Figure 3:**
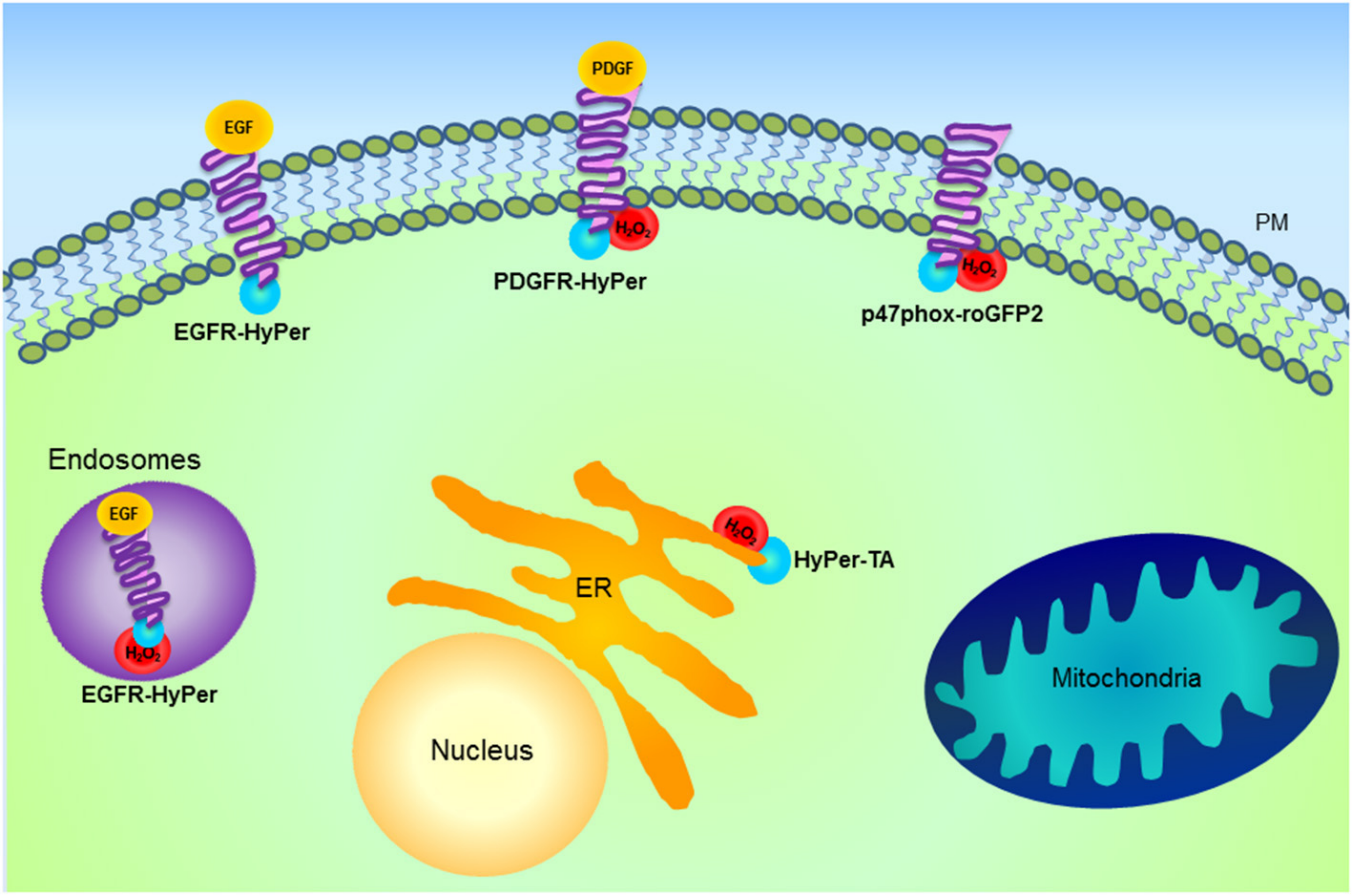
**Schematic representation of H_2_O_2_ microdomains within different subcellular compartments**. Genetically encoded probe HyPer (blue) fused with different proteins including epidermal growth factor receptor (EGFR), platelet-derived growth factor receptor (PDGFR), Nox and tail anchor of protein tyrosine phosphatase 1B (HyPer-TA) is shown. HyPer fusion proteins allow the visualization of focal H_2_O_2_ formation. Activation of EGFR is associated with H_2_O_2_ microdomains formation (red) on the endosomes, while PDGFR activation generates H_2_O_2_ microdomains at the plasma membrane (PM) and only after prolonged incubation on the endosomes. Activation of both receptors results in the oxidation of HyPer-TA, localized at the cytoplasmic side of the endoplasmic reticulum (ER) membrane. This suggests that following agonist stimulation, there is specific activation of PM- or ER-associated Nox pools. Besides the indicated approaches for microdomain investigation, as detailed in the text, HyPer targeted to various cellular compartments has been used to characterize spatio-temporal differences in H_2_O_2_ formation. For instance, in the case of mitochondria information has been obtained by specific HyPer targeting to the matrix or intermembrane space, while microdomain characterization has not yet been exploited.

Redox status within the compartments can also be heterogeneous with localized areas of ROS production and others with more reducing environment. For instance, it is possible that when H_2_O_2_ is produced locally it only oxidizes a few redox sensor molecules and then diffuses so that the signal gets diluted in the cytosol. Fusion of HyPer to different proteins localized on the cytoplasmic face of the plasma membrane (EGFR, PDGFR), endosomes and the ER membrane allows visualization of sites of focal ROS formation within compartments (Lukyanov and Belousov, 2014). Activation of EGFR was associated with H_2_O_2_ microdomains (due to Nox activation) on the endosomes and the cytoplasmic side of the ER membrane, but no H_2_O_2_ formation was observed associated with the plasma membrane. PDGFR activation instead generated H_2_O_2_ microdomains at the plasma membrane and only after prolonged incubation on the endosomes, suggesting that a specific plasma membrane-residing Nox pool was activated (Mishina et al., 2011) (Figure 3). Importantly, the diffusion of H_2_O_2_ within the cytoplasm was restricted to less than a 1 μm radius.

Another mechanism of signal transduction linking ROS and tyrosine phosphorylation is mediated through regulation of phosphatase activity. PTPs (and other structurally related phosphatases, such as PTEN) contain cysteine residues critical for enzyme activity, whose oxidation leads to their reversible inactivation (Tonks, 2005; Ostman et al., 2011). The prerequisite for signaling is reversibility of this modification, however it is not known whether localized production of ROS can account for specific inactivation of one phosphatase (or a pool) over another. Fusion of HyPer to the tail anchor of PTP1B (HyPer-TA) resulted in the expression of the sensor on the cytoplasmic surface of the ER membrane (as is the case with endogenously expressed PTP1B) (Mishina et al., 2011). Indeed, following EGFR or PDGFR activation HyPer-TA became oxidized with a different temporal pattern than described above for EGFR- or PDGFR-HyPer, suggesting that localized formation of ROS in vicinity of PTP1B is necessary for its specific activation and agonist-induced signal transduction.

Whether microdomains of ROS formation can occur also in mitochondria, for instance, on the outer/inner leaflet of the outer mitochondrial membrane, IMS or in the matrix remains to be established. So far, only a few studies attempted to elucidate this concept. roGFPs or HyPer targeted to the mitochondrial matrix, IMS, and cytosol showed that exposure of the cells to paraquat or MPP^+^ resulted in oxidation in the mitochondrial matrix that preceded the one in the cytosol, but did not lead to oxidative stress in the IMS (Rodriguez-Rocha et al., 2013). Rotenone instead, led to an early increase in H_2_O_2_ in the IMS and mitochondrial matrix that was later followed by the cytosolic compartment (Malinouski et al., 2011). Moreover, exposure of smooth muscle cells to hypoxia led to redox changes in the IMS and cytosol, but not in the mitochondrial matrix (Waypa et al., 2010). This suggests that areas of localized ROS formation or microdomains might exist within mitochondria, but further studies are necessary in this regard.

## Conclusions

The available evidence shows that each compartment within the cell has different redox characteristics that are in line with the function of each organelle. ROS are generated in different compartments as part of normal metabolic function and may act as signaling molecules. However, depending on the intensity and duration, these redox signals can also become damaging, triggering, and participating in processes that lead to cell death. Although more reducing compared to other compartments, mitochondria present high rate of ROS formation and a number of ROS sources. Each compartment in the cell is characterized by redox signaling involved in a variety of biological processes, both physiological and pathological and it is likely that a cross-talk exists, by which ROS formation within one compartment can trigger their formation in another thereby amplifying overall oxidative stress. Development of novel tools to measure compartmentalized ROS formation and redox status displays several advantages: (1) it allows for better characterization of ROS signals in compartments and microdomains, and (2) provides an explanation for antioxidants failure in treating several pathologies. The unsuccessful outcome of several clinical trials evaluating the therapeutic potential of antioxidants for the treatment of heart failure raised questions regarding the importance of ROS or oxidative stress. Taking into account data available and given the importance of ROS both in physiology and pathology, we propose that inhibition of specific processes that generate ROS rather than general antioxidants administration may prove as a successful therapeutic strategy.

### Conflict of interest statement

Nina Kaludercic and Fabio Di Lisa have a pending patent application entitled “Treatment of heart failure and associated conditions by administration of monoamine oxidase inhibitors,” number: 20090286883. The authors declare that the research was conducted in the absence of any commercial or financial relationships that could be construed as a potential conflict of interest.
